# The ability of patients with Parkinson’s disease to recognize masked faces during the COVID-19 pandemic

**DOI:** 10.1590/1980-5764-DN-2021-0117

**Published:** 2022-08-15

**Authors:** Sabiha Tezcan Aydemir, Müge Kuzu Kumcu, Nazlı Durmaz Çelik, Batuhan Bakirarar, Serhat Özkan, Muhittin Cenk Akbostancı

**Affiliations:** 1 Dr. Nafiz Körez Sincan Government Hospital, Neurology Clinic, Ankara, Turkey.; 2 Lokman Hekim University, Faculty of Medicine, Department of Neurology, Ankara, Turkey.; 3 Eskişehir Osmangazi University, Faculty of Medicine, Department of Neurology, Eskisehir, Turkey.; 4 Ankara University, Faculty of Medicine, Department of Biostatistics, Ankara, Turkey.; 5 Ankara University, School of Medicine, Department of Neurology, Ankara, Turkey.; 6 Ankara University, Department of Interdisciplinary Neuroscience, Ankara, Turkey.

**Keywords:** Parkinson Disease, COVID-19, Facial Recognition, Doença de Parkinson, COVID-19, Reconhecimento Facial

## Abstract

Patients with Parkinson’s disease (PwP) have face recognition difficulties.

**Objective:**

This study aimed to evaluate the difficulties of PwP in recognizing masked faces during the COVID-19 pandemic.

**Methods:**

A total of 64 PwP, 58 age-matched older healthy controls (OHCs), and 61 younger healthy controls (YHCs) were included in the study. The Benton Face Recognition Test - short form (BFRT-sf) and the 13-item questionnaire on face recognition difficulties due to masks during the pandemic developed by the authors were applied to all three study groups.

**Results:**

Both the PwP and OHC groups scored worse in BFRT-sf when compared with the YHC group (p<0.001 and p<0.001, respectively). The number of those who had difficulty in recognizing people seen every day and the number of those who asked people to remove their masks because they did not recognize them were higher in the PWP group (p=0.026 and p=0.002, respectively). The number of individuals who looked at the posture and gait of people when they did not recognize their masked faces and those who stated that this difficulty affected their daily lives were higher in the OHC group (p=0.002 and p=0.009, respectively). The number of participants whose difficulty in recognizing masked faces decreased over time was higher in the YHC group (p=0.003).

**Conclusions:**

The PwP group demonstrated similar performance to their peers but differed from the YHC group in recognizing masked faces. Knowing difficulties experienced by elderly people in recognizing people who are masked can increase awareness on this issue and enhance their social interaction in pandemic conditions through measures to be taken.

## RESUMO.

Pacientes com doença de Parkinson (PcP) têm dificuldades de reconhecimento facial.

**Objetivo:**

Avaliamos as dificuldades de PcP em reconhecer rostos mascarados durante a pandemia de COVID-19.

**Métodos:**

Incluímos 64 PcP, 58 controles saudáveis ​​mais velhos (CSVs) pareados por idade, 61 controles saudáveis mais jovens (CSJs) no estudo. O *Benton Face Recognition Test-short form* (BFRT-sf) e o questionário de 13 itens sobre dificuldades de reconhecimento facial devido a máscaras durante a pandemia desenvolvido pelos autores foram aplicados a todos os três grupos de estudo.

**Resultados:**

Ambos os grupos PcP e CSV tiveram pior pontuação no BFRT-sf quando comparados com o grupo CSJ (p<0,001 e p<0,001, respectivamente). O número daqueles que tiveram dificuldade em reconhecer as pessoas atendidas todos os dias e o número daqueles que pediram para as pessoas retirarem suas máscaras por não as reconhecer foram maiores no grupo PcP (p=0,026 e p=0,002, respectivamente). O número de indivíduos que olharam para a postura e marcha das pessoas quando não reconheceram seus rostos mascarados e aqueles que afirmaram que essa dificuldade afetou seu cotidiano foi maior no grupo CSV (p=0,002 e p=0,009, respectivamente). O número de participantes cuja dificuldade em reconhecer rostos mascarados diminuiu ao longo do tempo foi maior no grupo CSJ (p=0,003).

**Conclusões:**

O grupo PcP demonstrou desempenho semelhante aos seus pares, mas diferiu do grupo CSJ no reconhecimento de rostos mascarados. Conhecer as dificuldades vivenciadas pelos idosos em reconhecer as pessoas mascaradas pode aumentar a conscientização sobre essa questão e potencializar sua interação social em condições de pandemia por meio de medidas a serem tomadas.

## INTRODUCTION

Efforts to decrease the transmission of the novel coronavirus (COVID-19) have resulted in the widespread use of masks, which has significantly affected our facial recognition abilities and thus our social interaction. When the lower portion of the face is obscured by a mask, holistic processing strategies, which constitute the hallmark of face perception^1^, are expected to be ineffective and replaced by feature-based processing^2^. Holistic processing is defined as an upper-level strategy that is sensitive to the configuration and distances between all of the sub-features of the face (i.e., eyes, eyebrows, nose, chin, and lips) and predominant in recognizing familiar faces, whereas feature-based processing is a more primitive strategy that is useful on detecting unfamiliar or partially visible faces^3^.

Patients with Parkinson’s disease (PwP) generally have multiple visual dysfunctions attributed to the dopaminergic deficit in the retina and frontostriatal circuit^4^ ^,^ ^5^. To comprehend the scope of visual processing disorders in PwP, face recognition ability is frequently evaluated^6^ ^,^ ^7^ ^,^ ^8^ ^,^ ^9^. While some studies have revealed a general impairment in facial recognition and perception according to the Benton Facial Recognition Test (BFRT) in PwP compared to their peers with no PD, other studies report impairment in more specific, specialized tasks such as detecting similarities between morphed faces and evaluating facial expression from photographs^9^ ^,^ ^10^. Studies have reported that elderly individuals experience difficulties using holistic processing strategies in face perception^11^ ^,^ ^12^. A similar situation may also be valid for PwP and they may experience more difficulties than their peers in tasks that prioritize holistic processing, such as facial expression recognition^7^. Feature-based processing used in face recognition has been found to be intact in PwP under laboratory conditions^7^ ^,^ ^12^; however, challenges experienced by this population during daily exposure to masked faces remain uncertain.

It is well documented that feature-based processing strategies can be utilized in facial recognition of masked individuals^2^. Obligation to use masks provides a valuable opportunity to evaluate the ecological validity of previous studies.

In this study, we aimed to evaluate difficulties experienced by PwP in recognizing masked faces that are now considered as the new norm of current daily life.

## METHODS

### Study participants

Ethical approval was obtained from the Local Ethics Committee (dated December 28, 2020, no. 2020/017). The scope of the study was explained to each participant and all individuals provided written informed consent for participation. A total of 64 PwP over 50 years of age participated in the study. All these patients met the clinical criteria of the United Kingdom Parkinson’s Disease Society Brain Bank^13^. Each patient’s disease severity was assessed with the Hoehn and Yahr scale^14^. All the patients were undergoing dopamine replacement therapy and were tested in their “on” state. Patients who met PD dementia criteria^15^ and those who scored below 24 points in the Mini-Mental State Examination (MMSE)^16^ were excluded.

A total of 58 older healthy controls (OHCs) were recruited from the caregivers of patients without PD or members of the hospital staff. They were matched with the PwP group in terms of age, sex, and education level (p=0.235, p=0.743, and p=0.881, respectively).

As previous studies reported that individuals aged 30-35 years were the most successful group in face recognition^17^, 61 younger healthy controls (YHC) of this age range were also recruited from the hospital staff or caregivers of patients without PD. None of the individuals in the OHC and YHC groups had any psychiatric or neurological disorder (including dementia) or a family history of PD.

The best-corrected visual acuity measurements of all the study participants were normal. In addition, it was ensured that none of the participants had any diagnosis of eye disease (e.g., cataracts, diabetic retinopathy, glaucoma, optic neuritis, and macular degeneration) to reduce the possibility that peripheral visual impairments could interfere with face perception.

### Study design and procedures

Global cognitive efficiency was evaluated using MMSE in participants who were older than 50 years of age. All the participants were administered the Benton Face Recognition Test - short form (BFRT-sf)^18^, in which the subject has to match different photographs of the same unfamiliar face by choosing among six photographs. Some trials include views of the face taken from different angles, different facial expressions, or under different lighting conditions (minimum score: 0, maximum score: 27; a higher score indicates better face recognition). In addition, all the participants were asked to respond to a questionnaire prepared by the authors of this study, Face Recognition Difficulties due to Mask Use during the Pandemic, which consisted of 13 items to evaluate difficulties in recognizing masked faces (Table 1). We aimed to evaluate the participants’ face recognition difficulties, compensation methods used to overcome this difficulty, and the impact of difficulty in recognizing masked faces on their daily lives.

**Table 1.** Face recognition difficulties due to mask use during the pandemic questionnaire and analysis of responses.

	Patients with Parkinson’s disease % (yes, agree/total)	Older healthy controls % (yes, agree/total)	Younger healthy controls % (yes, agree/total)	p-value
Q1. Difficulty recognizing masked faces Have you ever been unable to recognize people, even momentarily, because they were wearing a mask during the pandemic? YES NO	87.5 (56/64)	91.4 (53/58)	96.7 (59/61)	0.167*
For those who responded yes to the question above				
Q2. Difficulty recognizing people seen almost every day Have you ever been unable to recognize the face of someone you normally see almost every day while he/she was wearing a mask? (Family members, colleagues, housemates, etc.) YES NO	10.7 (6/56)	5.7 (3/53)	0.0 (0/59)	0.026*
Q3. Difficulty recognizing people seen two to three times a week Have you ever been unable to recognize someone you would normally see at least two to three times a week while he/she was wearing a mask? (Neighbors, neighborhood residents, shopkeepers, relatives, etc.) YES NO	28.6 (16/56)	26.4 (14/53)	20.3 (12/59)	0.571**
Q4. Difficulty recognizing people seen every two to three weeks Have you ever been unable to recognize someone you would normally see every two to three weeks while he/she was wearing a mask? (Family doctor, rarely seen friends, and relatives, etc.) YES NO	51.8 (29/56)	47.2 (25/53)	42.4 (25/59)	0.600**
Q5. Difficulty recognizing people seen less than once a month Have you ever been unable to recognize someone you would normally see less than once a month while he/she was wearing a mask? (Distant relatives, acquaintances living in another city, etc.) YES NO	85.7 (48/56)	84.9 (45/53)	89.8 (53/59)	0.705**
Utilization of alternative cues used in the presence of difficulty in recognizing masked faces				
Q6. Looks carefully at the eye region When I am unable to recognize a person wearing a mask, paying attention to their eyes allows me to recognize them. AGREE NOT AGREE	78.6 (44/56)	83 (44/53)	83.1 (49/59)	0.781**
Q7. Looks carefully at the head region When I am unable to recognize a person wearing a mask, I need to look at their hairstyle or head accessories (hat, headscarf, necklace, earrings, glasses, etc.) more carefully in order to recognize them. AGREE NOT AGREE	55.4 (31/56)	35.8 (19/53)	37.3 (22/59)	0.068**
Q8. Needs to hear the person’s voice When I am unable to recognize a person wearing a mask, I need to hear their voice in order to recognize them. AGREE NOT AGREE	58.9 (33/56)	54.7 (29/53)	39.0 (23/59)	0.078**
Q9. Looks carefully at the clothes When I am unable to recognize a person wearing a mask, I need to look at their clothes more carefully in order to recognize them. AGREE NOT AGREE	30.4 (17/56)	37.7 (20/53)	28.8 (17/59)	0.565**
Q10. Looks carefully at posture and gait When I am unable to recognize a person wearing a mask, I need to look at their gait, posture, and body shape in order to recognize them. AGREE NOT AGREE	53.6 (30/56)	60.4 (32/53)	30.5 (18/59)	0.004**
Q11. Needs the person to remove his/her mask I have asked a person to remove their mask because I was unable to recognize them at least once. AGREE NOT AGREE	37.5 (21/56)	22.6 (12/53)	10.2 (6/59)	0.002**
Impact of difficulty in recognizing masked faces in daily life and changes in face recognition ability over time				
Q12. Increased success in recognizing masked faces Do you think you have improved at recognizing people wearing masks since the beginning of the pandemic? YES NO	43.8 (28/64)	55.2 (32/58)	73.8 (45/61)	0.003**
Q13. Daily life affected due to difficulty in recognizing faces Does difficulty recognizing masked people negatively affect your daily life? YES NO	12.5 (8/64)	29.3 (17/58)	9.8 (6/61)	0.009**

Q: question. p<0.05 is considered significant; *One-way analysis of variance; **Kruskal-Wallis H test.

### Statistical analysis

All statistical analyses were performed using Statistical Package for the Social Sciences (SPSS) version 22 for Windows 11.5 (SPSS Inc., Chicago, IL, USA). For descriptive data, quantitative variables were expressed as mean±standard deviation and median (min-max) values, while categorical (qualitative) variables were expressed as percentages (frequency). Mean values were compared using Student’s t-test if the normal distribution assumptions were met, and the Mann-Whitney U test was used otherwise. The relationship between two categorical variables was compared with the chi-square test or Fisher’s exact test. The relationship between three categorical variables was compared with one-way analysis of variance or the Kruskal-Wallis H test. The Bonferroni-corrected Mann-Whitney U test was used for the paired comparisons of statistically significant results in the comparisons between the three groups, and the significance level was set at p<0.05.

## RESULTS

### Clinical characteristics

The clinical characteristics of the study groups are presented in Table 2.

**Table 2.** Clinical characteristics of the participants.

Variable		PwP group (n=64)	OHC group (n=58)	YHC group (n=61)
Sex (F/M)		29/35	28/30	36/25
Age (years)	Mean±SD Median (min-max)	61.5±6.5 61.0 (50.0-80.0)	59.9±8.0 60.5 (50.0-74.0)	32.7±1.7 33.0 (30.0-35.0)
Education (years)	Mean±SD Median (min-max)	8.4±4.5 8.0 (0.0-16.0)	8.8±5.1 7.0 (0.0-21.0)	14.6±3.4 15.0 (8.0-21.0)
Disease duration (years)	Mean±SD Median (min-max)	6.1±3.5 5.0 (2.0-17.0)	N/A	N/A
UPDRS III	Mean±SD Median (min-max)	30.8±13.3 31.0 (3.0-68.0)	N/A	N/A
Hoehn and Yahr staging	Mean±SD Median (min-max)	2.2±0.7 2.5 (1.0-4.0)	N/A	N/A
MMSE	Mean±SD Median (min-max)	28.3±1.9 28.0 (24.0-30.0)	28.5±1.8 29.0 (24.0-30.0)	N/A
BFRT-sf	Mean±SD Median (min-max)	18.3±2.4 18.5 (13.0-24.0)	18.4±2.8 19.0 (8.0-24.0)	21.0±2.2 21.0 (16.0-27.0)

Values are presented as mean**±**SD and median (min-max). PwP: patients with Parkinson’s disease; OHC: older healthy control; YHC: younger healthy control; F: female, M: male; SD: standard deviation; UPDRS III: Unified Parkinson’s Disease Rating Scale Part III motor score; MMSE: Mini-Mental State Examination; BFRT-sf: Benton Face Recognition Test - short form.

### Analysis of neuropsychological test data

The mean MMSE score was 28.2±1.4 (24.0-30.0) in the PwP group and 28.4±1.2 (24.0-30.0) in the OHC group (p=0.056). The mean BFRT-sf score was 18.3±2.4 (13.0-34.0) in the PwP group, 18.4±2.8 (min-max 8.0-24.0) in the OHC group, and 21.0±2.2 (min-max 16.0-27.0) in the YHC group (p<0.001). When the three groups were compared in terms of the BFRT-sf scores, there was no statistically significant difference between the PwP and OHC groups (p=1.000), but a statistically significant difference was observed between the PwP and YHC groups and between the OHC and YHC groups (p<0.001 and p<0.001, respectively).

### Analysis of questionnaire results

When the responses to the first question were evaluated, it was observed that 87.5% of the PwP, 91.4% of the OHCs, and 96.7% of the YHCs had experienced difficulty recognizing masked faces at least once during the pandemic (p=0.167). Only the individuals who had experienced difficulty recognizing masked faces (responded yes to question 1) were asked to answer questions 2 to 11 to assess helpful strategies they used to recognize masked individuals.

When the participants were evaluated in terms of difficulty recognizing masked people that they saw at different intervals, the PwP and OHC groups and the OHC and YHC groups were determined to have similar rates of difficulty recognizing people they saw every day (p=1.000 and p=0.309, respectively). However, the PwP group had a higher rate of individuals with this difficulty compared to the YHC group (p=0.036).

The rates of participants that observed posture and walking to help identify masked people when they could not recognize them were similar between the PwP and OHC groups (Bonferroni-corrected; p=1.000), while it was significantly higher in the YHC group compared to the remaining two groups (p=0.003 and p=0.036, respectively). The rate of individuals asking an unrecognized person to remove their mask to recognize them were similar between the PwP and OHC groups and between the YHC and OHC groups (p=0.273 and p=0.219, respectively) but significantly higher in the PwP group than in the YHC group (p=0.003).

The number of participants indicating that they became more successful at recognizing masked faces compared to the beginning of the pandemic was similar between the PwP and OHC groups and between the YHC and OHC groups (p=0.624 and p=0.102, respectively), but it was significantly higher in the YHC group compared to the PwP group (p=0.003).

The number of participants indicating that difficulty recognizing faces affected their daily lives was similar between the PwP and OHC groups and between the PwP and YHC groups (p=0.066 and p=1.000, respectively), but it was significantly higher in the OHC group compared to the YHC group (p=0.021). Table 1 presents the analysis of the participants’ responses to the questionnaire items.

## DISCUSSION

We approached the face recognition ability of PwP from a different perspective than the published data by evaluating the ability to recognize masked faces in daily life rather than performing an office-based assessment. With this approach, we aimed to understand difficulties experienced by PwP in this new challenge of being exposed to masked faces on a daily basis.

According to an online study conducted with younger healthy people, recognizing faces with a mask requires feature-based processing rather than holistic processing^2^. In studies related to face recognition abilities in PwP and in people older than 50 years, deterioration was shown from early stages in tasks involving the use of holistic processing strategies, such as the detection of facial expression. Feature-based processing, however, is generally intact in PwP^7^ ^,^ ^12^ ^,^ ^19^ ^,^ ^20^. Therefore, it may be considered that none of our groups was at a disadvantage in terms of their ability to recognize masked faces. However, the data of our questionnaire produced slightly different results than expected.

A previous study that used eye-tracking devices showed that directing attention more frequently toward the eye region increased the accuracy of recognizing members of the Caucasian people. Considering the large population of the Caucasian people in our society and the data indicating that looking at the eye area to identify people improves recognition accuracy in this group^21^, it can be deduced that directing attention toward the eye region will minimize difficulty identifying a familiar masked face. According to the results of our questionnaire, the need to direct attention to visual clues other than the eye area was more common in the >50 years group. This may indicate that people over the age of 50 years have more difficulty than younger people in recognizing masked faces and are, therefore, at a disadvantage compared to the later in such a task requiring feature-based processing. Although previous studies have stated that feature-based processing is intact in PwP and OHCs^7^ ^,^ ^12^, our findings indicate that this may differ in daily life.

Derya et al. reported that the PwP performed similar to their peers in detecting facial expression shown on a video whereas they were at a disadvantage when asked to recognize facial expressions from photographs in a laboratory task^22^. When this finding is evaluated together with the results of previous studies reporting a disconnection of the pathways extending from the prefrontal region to the ventral and dorsal pathways of visual processing in PwP^23^, the PwP performing similar to their peers in recognizing facial expressions in a more dynamic circumstance of a video may indicate that the dorsal pathway associated with dynamic processes is less affected in PD. In this regard, the parallel responses of our PwP and OHC groups to most items of the questionnaire may be related to our evaluation of face recognition in a dynamic process, similar to a video. Likewise, the parallel responses to the questionnaire and BFRT-sf scores among the individuals aged over 50 years in the OHC and PwP groups, which separates them from the YHC group, may also be associated with age-related degeneration.

In this study, the BFRT-sf scores of the PwP and OHC groups without dementia were similar, while the performance of both groups was worse compared to the younger group, which is consistent with data in the literature demonstrating that this test score decreases with age^18^.

In brief, when we examined the results of BFRT-sf and the 13-item questionnaire prepared for this study, the PwP and OHC groups displayed similar performance in the domain of recognizing masked faces during the pandemic and performed significantly worse than the YHC group. The rates of individuals with difficulty recognizing people they saw every day and asking an unrecognized person to remove their mask to recognize them were higher in the PwP group than in the YHC group, but there was no statistically significant difference between the OHC and YHC groups. These findings are consistent with the results of another study that compared PwP, OHC, and YHC groups in terms of recognizing facial expressions on a video^22^. However, in contrast to that study evaluating tasks using holistic processing, we assessed a challenging aspect of daily life that emphasizes feature-based processing, which is reportedly intact in individuals over the age of 50 years. In this regard, our study is valuable in terms of revealing how tests performed in the laboratory and daily life experiences can differ. In this way, it is important to acknowledge difficulties experienced by elderly people with or without PD in masked face recognition and improve conditions that facilitate their social life in prolonged pandemic conditions.

A limitation of this study is that we did not evaluate the presence of depression and anxiety disorders among the participants, which could affect their facial recognition performance. Since there is no validated scale or visual test evaluating masked face recognition, our data based on subjective opinion of the participants collecting by a questionnaire, which is a pitfall of the study. However, the reason for these was to shorten their duration of hospital stay to prevent the possible transmission of COVID-19 disease among the researchers and subjects.

In conclusion, the widespread use of facemasks due to the pandemic poses a new challenge in face recognition. According to the results of BFRT-sf and the participants’ responses to the Face Recognition Difficulties due to Mask Use during the Pandemic Questionnaire, the PwP group demonstrated similar performance to their peers but differed from the younger people in recognizing masked faces requiring feature-based processing.

Various strategies may be useful to prevent elderly people from decreasing their social interaction due to the use of facemasks. For example, web-based masked face recognition tests can be designed for training as social cognitive rehabilitation. In addition, the use of transparent masks can be an alternative method to improve interpersonal communication.
